# Nucleolin modulates compartmentalization and dynamics of histone 2B-ECFP in the nucleolus

**DOI:** 10.1080/19491034.2018.1471936

**Published:** 2018-06-26

**Authors:** Ayantika Sen Gupta, Gaurav Joshi, Sumit Pawar, Kundan Sengupta

**Affiliations:** Biology, Indian Institute of Science Education and Research (IISER), Pune, India

**Keywords:** Nucleolus, H2B, nucleolin, lamin, nucleus, rRNA

## Abstract

Eukaryotic cells have 2 to ​3 discrete nucleoli required for ribosome synthesis. Nucleoli are phase separated nuclear sub-organelles. Here we examined the role of nuclear Lamins and nucleolar factors in modulating the compartmentalization and dynamics of histone 2B (H2B-ECFP) in the nucleolus. Live imaging and Fluorescence Recovery After Photobleaching (FRAP) of labelled H2B, showed that the depletion of Lamin B1, Fibrillarin (FBL) or Nucleostemin (GNL3), enhances H2B-ECFP mobility in the nucleolus. Furthermore, Nucleolin knockdown significantly decreases H2B-ECFP compartmentalization in the nucleolus, while H2B-ECFP residence and mobility in the nucleolus was prolonged upon Nucleolin overexpression. Co-expression of N-terminal and RNA binding domain (RBD) deletion mutants of Nucleolin or inhibiting 45S rRNA synthesis reduces the sequestration of H2B-ECFP in the nucleolus. Taken together, these studies reveal a crucial role of Nucleolin-rRNA complex in modulating the compartmentalization, stability and dynamics of H2B within the nucleolus.

## Introduction

The nucleus houses chromatin and several non-membranous nuclear bodies involved in transcription [1], splicing [2] and nuclear transport [3]. The absence of membranes within the nucleus facilitates dynamic but regulated exchange of molecules between nuclear bodies and chromatin [4]. The import and sequestration of protein and RNA into nuclear bodies modulates their nucleoplasmic concentration and function. The nucleolus is the largest nuclear sub-organelle essential for ribosome biogenesis [5]. The nucleolus also functions as a stress-sensing compartment that sequesters oncoproteins such as BRCA1 and regulators of p53, that are released into the nucleoplasm upon DNA damage [6,7], while, HSP70 and VHL proteins are immobilized in the nucleolus during thermal stress and acidosis respectively [8]. Key mechanisms of protein sequestration into the nucleolus are (i) interaction of proteins with resident nucleolar factors such as Nucleolin and Nucleophosmin [9–11] (ii) nucleolar localization signal (NoLS) enriched in lysine and arginine rich repeats [12] and (iii) interaction of proteins with non-coding RNA transcribed from intergenic sequence of the rDNA [8]. Mass spectrometric analyses of nucleolar extracts identified ~4500 proteins, which include isoforms of each histone family – H1, H2, H3, H4 and histone-modifying enzymes [13].

Electron microscopy reveals a remarkable tripartite structure of the nucleolus with a central Fibrillar Center (FC), surrounded by the Dense Fibrillar Component (DFC) and the Granular Component (GC). Such an organization facilitates ribosome biogenesis [5,14]. The nucleolus partitions into sub-compartments as a result of the separation of immiscible phases of Fibrillarin and Nucleophosmin [15]. Nucleolar structure is maintained by ongoing rDNA transcription, as its inhibition by Actinomycin D induces nucleolar segregation [16]. Nucleolar structure is also regulated by Nucleolin – one of the most abundant proteins of the GC [17]. Nucleolin has diverse roles in rDNA transcription, ribosome biogenesis [18], DNA damage repair [19] and regulation of apoptosis [20]. *In vitro* studies implicate Nucleolin as a histone chaperone with FACT-like activity, which regulates SWI-SNF function and ACF chromatin remodelers [21]. Nucleolin has a High Mobility Group (HMG)-like N-terminal domain with four acidic stretches of glutamate and aspartate residues, interspersed with basic lysine residues [22]. The acidic stretches interact with histone H1 while the basic residues interact with DNA [22]. Nucleolin also has four central RNA binding domains (RBD1-4) and a C-terminal GAR (Glycine Arginine Rich) domain. The RNA binding domain specifically binds to a 5′ external transcribed sequence (ETS) site on nascent ribosomal RNA. The GAR domain of Nucleolin binds specifically to DNA and non-specifically to RNA, while the RBDs confer specificity to RNA binding [23–25]. ChIP-Seq analysis reveals the recruitment of Nucleolin to sites of DNA damage, resulting in the eviction of histones – H2A and H2B thereby allowing access to the DNA double strand break repair machinery [19]. H2B has been detected in the nucleoli of Bovine liver cells and chicken erythrocytes using antibodies raised against its first 58 amino acids [26]. Localization of H2B in the nucleolus is attributed to stretches of basic amino acid residues (KKRKRSRK), similar to the NoLS motifs: (R/K)(R/K)X(RK) or (R/K)X(R/K)(R/K) [27].

Here we show the RNA-dependent function of Nucleolin in modulating the localization, dynamics and retention of Histone 2B (H2B-ECFP) in the nucleolus.

## Results

### Histone 2B (H2B) compartmentalizes in the nucleolus

**Figure 1.**
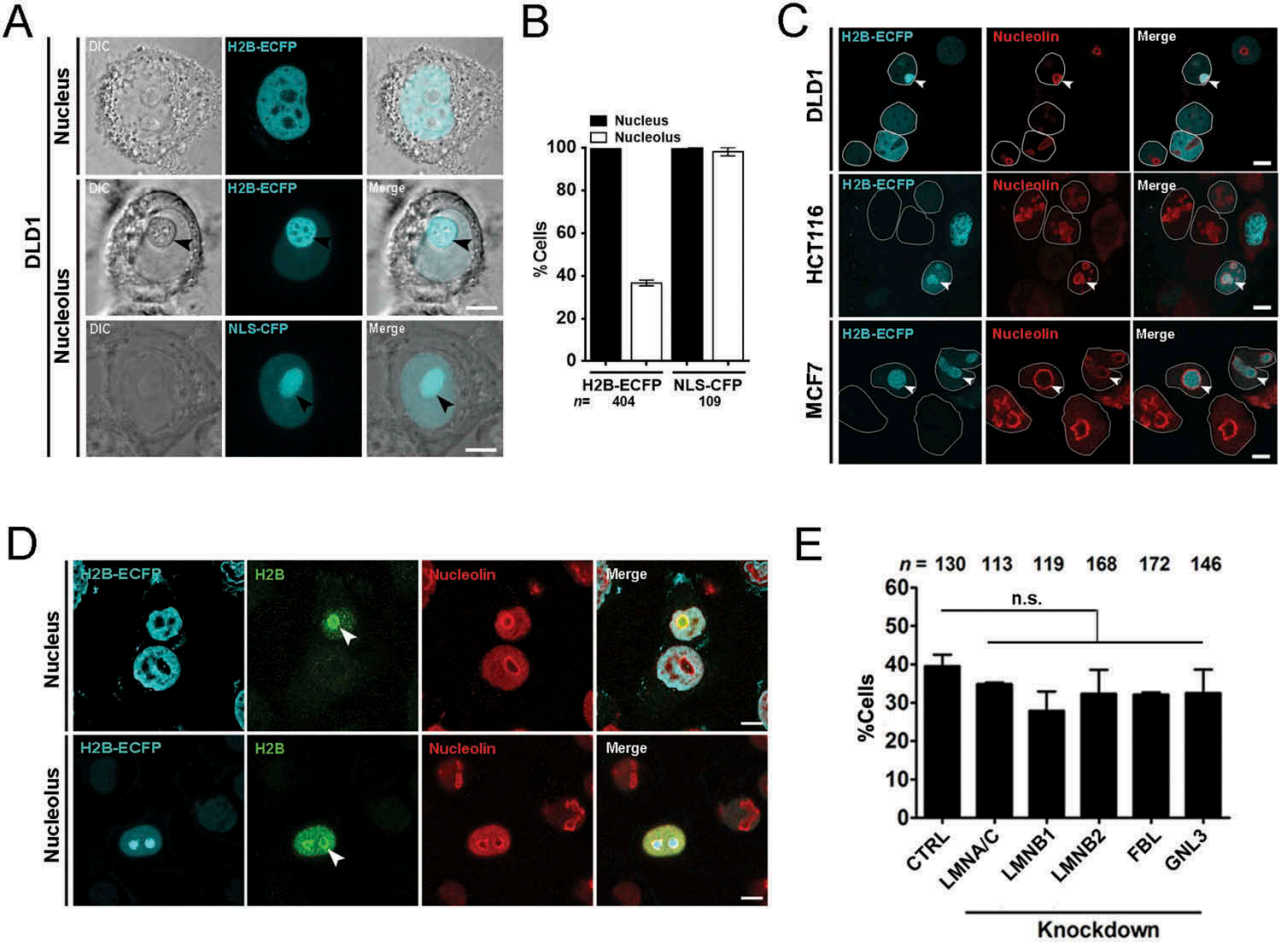
Histone 2B-ECFP localizes in the nucleolus. (a) H2B-ECFP is distinctly localized in the nucleoplasm and the nucleolus. Top panel: nucleoplasmic localization of H2B-ECFP, Middle panel: localization of H2B-ECFP in the nucleolus (black arrowhead). Bottom panel: NLS-CFP localizes to the nucleoplasm and the nucleolus (black arrowhead). Scale bar ~5 μm. (b) All transfected cells show H2B-ECFP in the nucleoplasm, while ~40% of these cells harbor H2B-ECFP in the nucleolus. All cells show NLS-CFP in the nucleoplasm, while ~98% cells show NLS-CFP in the nucleolus, n = number of nuclei, data compiled from N = 2 independent biological replicates. (c) Immunostaining of Nucleolin marks nucleoli with H2B-ECFP in DLD1, HCT116 and MCF7 cells (white arrows). White outline demarcates single nucleus, scale bar ~5 μm. (d) Cells transfected with H2B-ECFP were immunostained with anti-histone 2B antibody and anti-nucleolin antibody to demarcate the nucleolus (white arrows), anti-histone 2B antibody detects both transfected and endogenous H2B in the nucleolus. (e) Independent knockdowns of Lamin A/C, B1, B2, FBL and GNL3 do not affect the extent of nucleolar localization of labeled H2B-ECFP, n = number of nuclei, data compiled from N = 3 independent biological replicates, error bars: SEM. Student’s t-test, p > 0.05 (n.s: not significant).

The nucleolus is the largest nuclear sub-organelle and is essential for ribosomal RNA (rRNA) and protein synthesis [28]. However, the mechanisms that regulate the sequestration of proteins within the nucleolus remain unclear. For instance, overexpressed H2B is sequestered in the nucleolus [27]. Here we sought to investigate the mechanisms that modulate the sequestration and dynamics of H2B-ECFP in the nucleolus. We transfected H2B-ECFP into DLD1 colorectal cancer cells and found that although H2B-ECFP localizes in the nucleoplasm of all cells, a significant sub-population of cells (~40%) show H2B-ECFP in the nucleolus (Figure 1(a,b)). While, the Nuclear Localization Signal (NLS) sequence tagged with CFP localizes in the nucleolus of nearly all transfected cells (~98%) (Figure 1(a,b)). We surmise that the relatively small NLS-CFP freely diffuses into the nucleolus, while the nucleolar localization of H2B-ECFP in a sub-population of ~40% cells, is potentially guided by additional interactions with nucleolar factors. H2B-ECFP localizes in the nucleolus of diverse cancer cell lines such as HCT116 (colorectal cancer cell line), MCF7 (breast cancer cell line) as well as DLD1 cells (Figure 1(c)). In addition to visualizing nucleolar localization of overexpressed H2B-ECFP, we found that endogenous H2B also localizes in the nucleolus as revealed by immunofluorescence assays (Figure 1(d)).

Lamin A regulates nuclear histone dynamics, while Lamin B1 and Lamin B2 modulate nucleolar organization and function [29–31]. We asked if nuclear Lamins or nucleolar factors i.e fibrillarin (FBL) and nucleostemin (GNL3), modulate the compartmentalization of H2B-ECFP in the nucleolus (Figure 1(e)). We independently knocked down nuclear Lamins, Fibrillarin (FBL) and Nucleostemin (GNL3) in DLD1 cells. Interestingly, knockdown of Lamin A/C (LMNA/C), Lamin B1 (LMNB1), Lamin B2 (LMNB2) or nucleolar factors – Fibrillarin (FBL) and Nucleostemin (GNL3) did not significantly affect the extent of H2B-ECFP localization within the nucleolus (Figure 1(e)). Taken together, these results suggest that the nucleolar localization of H2B-ECFP is unaffected by the depletion of Lamins or nucleolar factors such as FBL and GNL3.

### Lamin B1 enhances mobility of H2B-ECFP in the nucleolus

We sought to investigate the dynamics of fluorescently labelled H2B in the nucleolus and nucleus by Fluorescence Recovery After Photobleaching (FRAP) (Fig. S1A, B). Interestingly, photobleaching H2B in the nucleolus showed a significantly higher mobile fraction (M.F. ~40%) as compared to the nuclear sub-pool (M.F ~18%) (Fig. S1C). While NLS-CFP showed complete and immediate recovery further underscoring its ability to freely diffuse into the nucleus as well as the nucleolus (Fig. S1D, E).

**Figure 2.**
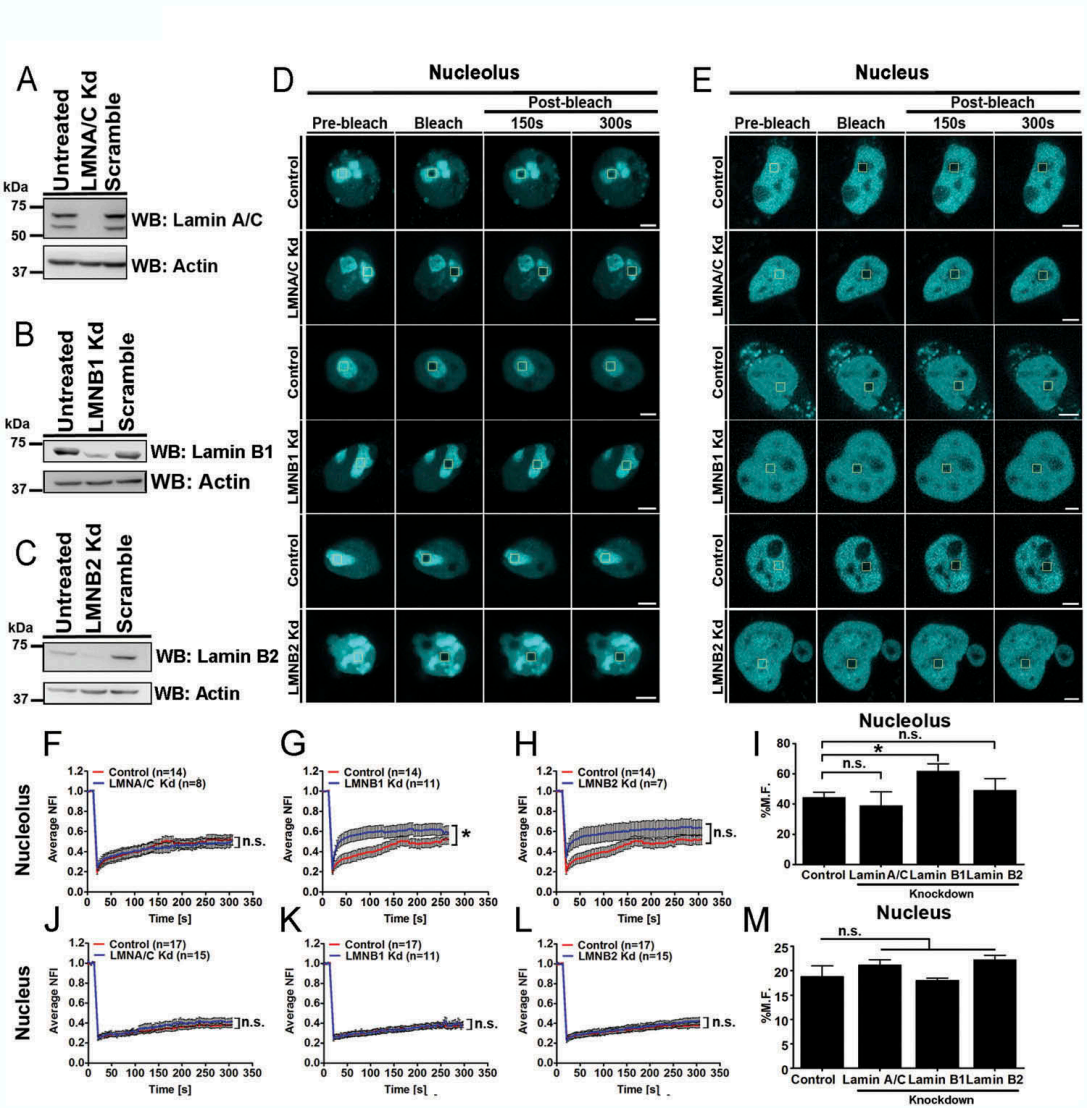
Lamin B1 depletion enhances H2B–ECFP mobility in the nucleolus. (a–c) Western blots of whole cell lysates prepared from (a) LMNA/C (b) LMNB1 and (c) LMNB2 knockdown. Controls: Untreated, scramble siRNA. Loading control: Actin. (d–e) Representative images showing FRAP of H2B-ECFP in (d) nucleolus and (e) nucleus of control, LMNA/C Kd, LMNB1 Kd and LMNB2 Kd cells. Yellow box represents bleached ROI. Scale bar ~5 µm. (f–h) Normalized fluorescence recovery curves comparing recovery of H2B-ECFP in the nucleolus of control, (f) LMNA/C Kd (g) LMNB1 Kd and (h) LMNB2 Kd cells. (i) Relative mobile fractions of H2B-ECFP in the nucleolus as calculated from (f–h), showing increased mobility of H2B-ECFP in the nucleolus upon Lamin B1 Kd. (j–l) Normalized fluorescence recovery curves of H2B-ECFP in the nucleus of control, (j) LMNA/C Kd (k) LMNB1 Kd (l) LMNB2 Kd cells. (m) Relative mobile fractions of H2B-ECFP in the nucleus as calculated from (j–l). Lamin knockdown does not affect H2B-ECFP mobility in the nucleus, n = number of nuclei, data compiled from N = 3 independent biological replicates, error bars: SEM in recovery curves and bar graph. Student’s t-test, *p < 0.05.

Since, nuclear Lamins maintain the structural and functional integrity of the nucleus [32,33], we asked if Lamins regulate H2B-ECFP dynamics. We performed siRNA mediated knockdown followed by immunoblotting, which showed ~70% depletion of Lamins in DLD1 cells (Figure 2(a–c)). We next performed FRAP of H2B-ECFP in the nucleolus and the nucleus respectively upon Lamin depletion (Figure 2(d,e)). Interestingly, Lamin A/C knockdown did not affect H2B-ECFP dynamics in the nucleolus (M.F. ~38.77%) (Figure 2(f,i), Table 1), while Lamin B1 knockdown showed a significant increase in the mobile fraction of H2B-ECFP (M.F ~61.63%) (Figure 2(g,i), Table 1). Lamin B2 knockdown also showed a marginal increase in H2B-ECFP mobility (~48.98%) (Figure 2(h,i), Table 1). In sharp contrast, Lamin knockdowns did not significantly alter H2B-ECFP mobility in the nucleus (Figure 2(j–m), Table 1). Of note, neither endogenous nor overexpressed levels of H2B were altered upon Lamin knockdowns (Fig. S1F). Taken together, Lamin B1 knockdown enhances H2B-ECFP mobility in the nucleolus.

**Table 1.** Mobile fractions of H2B-ECFP calculated from fluorescence recovery after photobleaching (FRAP) in the nucleus and nucleolus of DLD1 cells in Control, knockdowns of Lamin A, B1, B2, Fibrillarin, Nucleostemin, Nucleolin; and Nucleolin overexpression. Error represents SEM, p-value <0.05 considered significant as calculated from unpaired Students t-test (two-tailed).

	H2B-ECFP Mobile fraction (%) ± S.E.M.	
	Nucleus	Nucleolus
Control	18.79 ± 2.19 (n = 17)	44.3 ± 3.44 (n = 16)
Lamin A Kd	21.14 ± 1.13 (n = 15, p = 0.62)	38.77 ± 9.27 (n = 8, p = 0.59)
Lamin B1 Kd	18.01 ± 0.47 (n = 11, p = 0.77)	61.63 ± 5.05 (n = 11, *p = 0.015)
Lamin B2 Kd	22.25 ± 0.87 (n = 15, p = 0.39)	48.98 ± 7.9 (n = 7, p = 0.599)
Fibrillarin Kd	17.11 ± 2.22 (n = 13, p = 0.59)	68.81 ± 8.09 (n = 7, *p = 0.02)
Nucleostemin Kd	12.75 ± 0.46 (n = 13, *p = 0.03)	69.44 ± 3.16 (n = 11, **p = 0.0058)
Nucleolin GFP OE	–	71.76 ± 3.43 (n = 17, **p = 0.0048)

*p < 0.05, **p < 0.01.

### Fibrillarin (FBL) and Nucleostemin (GNL3) modulate H2B-ECFP dynamics within the nucleolus

**Figure 3.**
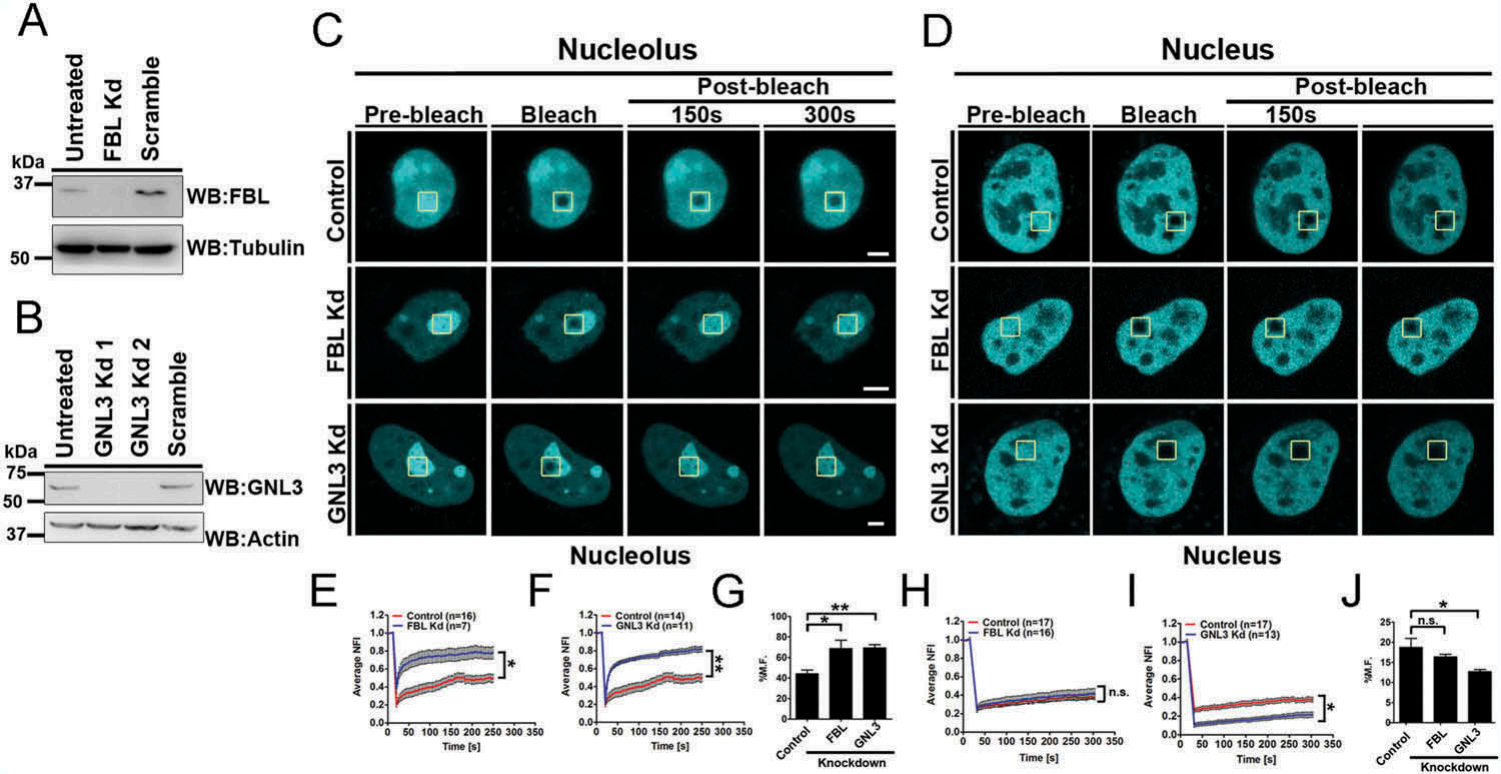
Depletion of Fibrillarin (FBL) and Nucleostemin (GNL3) enhances H2B–ECFP mobility in the nucleolus. (a, b) Western blots of whole cell lysates prepared from DLD1 cells upon knockdown of (a) Fibrillarin (FBL) (b) Nucleostemin (GNL3), Controls: untreated and respective scramble siRNA treated cells. Loading controls: Tubulin, Actin. (c, d) FRAP of H2B-ECFP in the (c) nucleolus and (d) nucleus of control, FBL and GNL3 Kd cells, respectively. Yellow box represents bleached ROI. Scale bar ~5 µm. (e, f) Normalized fluorescence recovery curves comparing recovery of H2B-ECFP in the nucleolus of control (e) FBL Kd and (f) GNL3 Kd cells. (g) Relative mobile fractions of H2B-ECFP in the nucleolus as calculated from (e, f). (h, i) Normalized fluorescence recovery curves comparing recovery of H2B-ECFP in the nucleus of control, (h) FBL Kd (i) GNL3 Kd cells. (j) Relative mobile fractions of H2B-ECFP in the nucleus as calculated from (h, i), n = number of nuclei, data from N = 3 independent biological replicates, error bars: SEM in recovery curves and bar graph. Student’s t-test, *p < 0.05, **p < 0.01.

We sought to examine if bonafide nucleolar proteins of the DFC and GC regions of the nucleolus – Fibrillarin (FBL) and Nucleostemin (GNL3), respectively, modulate H2B-ECFP dynamics in the nucleolus (Figure 3) [34,35]. We performed siRNA mediated knockdown of FBL and GNL3 in DLD1 cells, followed by western blotting, which showed ~80% depletion (Figure 3(a,b)). We next examined H2B-ECFP dynamics in the nucleolus and nucleus respectively upon FBL and GNL3 depletion (Figure 3(c,d)). FBL and GNL3 knockdown significantly increased the mobility of H2B-ECFP in the nucleolus (FBL Kd: M.F ~68.81%), (GNL3 Kd: M.F ~69.44%) (Figure 3(e–g), Table 1). Interestingly photobleaching the nuclear sub-pool of H2B-ECFP, showed a marginal decrease in its nuclear dynamics (FBL Kd: M.F ~17.11%) (Figure 3(h,j), Table 1), while Nucleostemin (GNL3) depletion showed a significant decrease in the mobile fraction of H2B-ECFP (GNL3 Kd: M.F ~12.75%) in the nucleus (Figure 3(i,j), Table 1). Taken together, these results reveal that Fibrillarin and Nucleostemin depletions modulate the dynamics of H2B-ECFP in the nucleolus, further underscoring the role of FBL and GNL3 in maintaining the microenvironment and stability of the nucleolus.

### Nucleolin modulates compartmentalization of H2B-ECFP in the nucleolus

**Figure 4.**
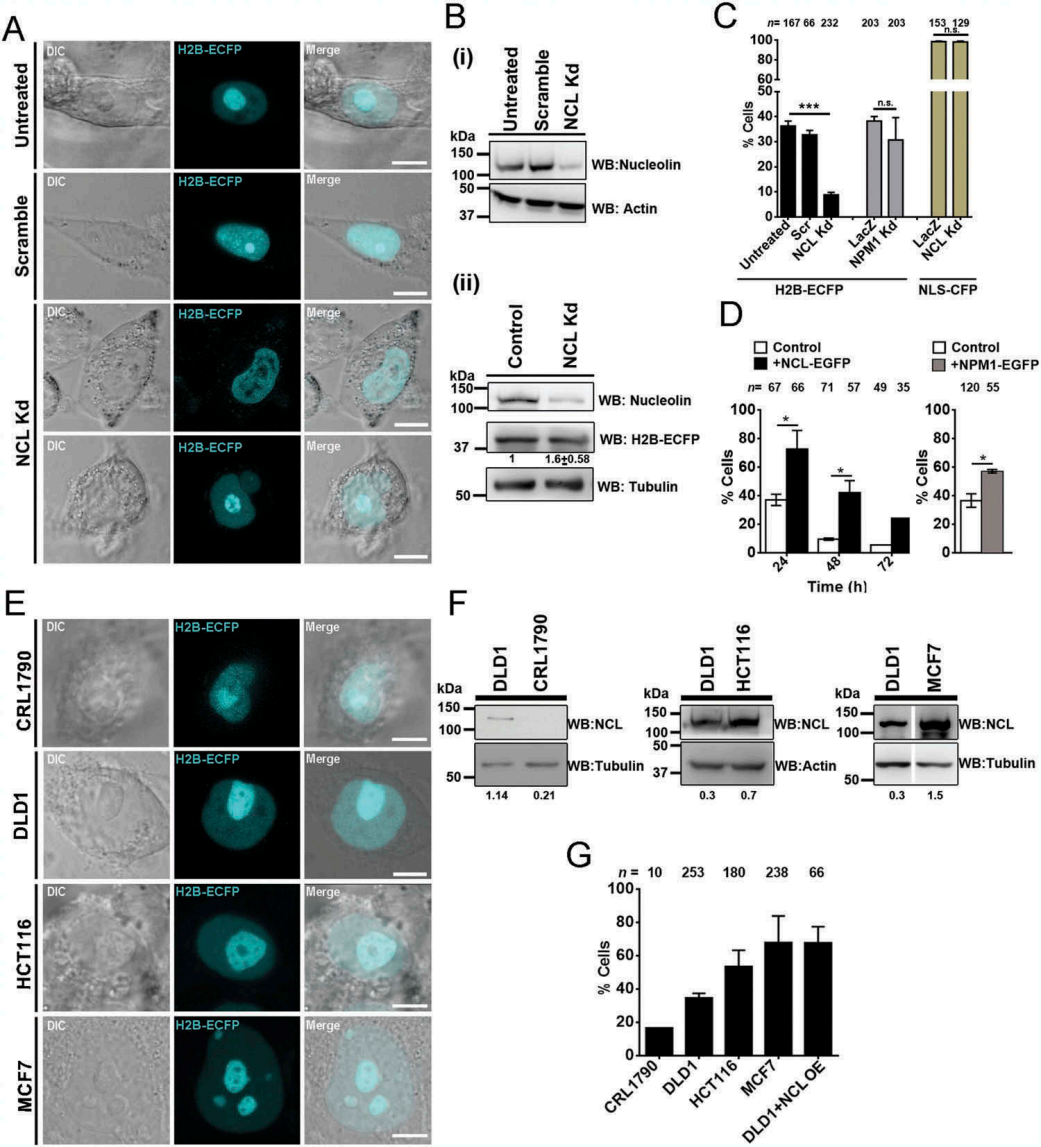
Nucleolin levels modulate compartmentation of H2B-ECFP in the nucleolus. (a) Representative images from live imaging of H2B-ECFP upon Nucleolin knockdown (NCL Kd) in DLD1 cells. Controls: untreated and scrambled siRNA treated cells. Scale bar ~5 µm. (b) (i) Western blots performed on whole cell lysates to detect Nucleolin levels in untreated, scramble and NCL siRNA treated DLD1 cells. Loading control: Actin. (ii) H2B-ECFP expression is marginally increased upon Nucleolin knockdown. Loading control: Tubulin. (c) Percent cells showing nucleolar H2B-ECFP compartments upon NCL Knockdown (Kd), n = number of nuclei, data from N = 3 independent biological replicates, error bars: SEM. Percent cells showing nucleolar H2B-ECFP upon NPM1 knockdown, n = number of nuclei, data from N = 2 independent biological replicates, error bars: SD. Percent cells showing nucleolar NLS-CFP upon NCL Kd, n = number of nuclei, data from N = 2 independent biological replicates, error bars: SD. Student’s t-test, ***p < 0.001. (d) Nucleolar H2B-ECFP upon NCL-GFP overexpression. DLD1 cells transfected with H2B-ECFP only (control, white bars) and co-transfected with H2B-ECFP and NCL-GFP (+NCL-GFP, black bars) imaged at intervals of 24, 48, and 72 h post transfection. Percent cells showing nucleolar H2B-ECFP upon 24 h of NPM1-GFP overexpression, n = number of nuclei, data from two independent biological replicates, N = 2, error bars: SD. (e) Representative images from live imaging H2B-ECFP transfected CRL1790, DLD1, HCT116 and MCF7 cells showing nucleolar localization of H2B-ECFP. Scale bar ~5 µm. (f) Western blots showing endogenous levels of NCL in CRL1790, DLD1, HCT116, and MCF7 cells. Loading controls: Tubulin, Actin. Intensity of Nucleolin normalized to loading control. (g) Extent of H2B-ECFP in the nucleolus in CRL1790, DLD1, HCT116, MCF7 cells and in DLD1 cells co-transfected with NCL-GFP, n = number of nuclei, data from two independent biological replicates, N = 2.

Nucleolin is a bonafide GC component protein that maintains nucleolar integrity and stability [36]. We asked if Nucleolin modulates the sequestration of H2B-ECFP in the nucleolus. We knocked down Nucleolin, followed by H2B-ECFP transfection into DLD1 cells (Figure 4(a,b)(i)). Interestingly, Nucleolin depletion revealed a striking reduction in the number of cells with H2B-ECFP in the nucleolus (<10%), as compared to control cells (~36%) (Figure 4(c)). H2B-ECFP expression was marginally higher in Nucleolin depleted cells (Figure 4(b)(ii)). This contrasts with Lamin, FBL and GNL3 depletion, which did not alter the extent of H2B-ECFP compartmentalization in the nucleolus (Figure 1(e)). The independent depletion of another nucleolar GC protein namely Nucleophosmin (NPM1), also showed a marginal reduction of H2B-ECFP in the nucleolus (~30%) as compared to control cells (~38%) (Figure 4(c), Fig. S2B). Decrease in nucleolar H2B-ECFP upon NPM1 knockdown, is consistent with the association between NPM1 and core, linker histones (H1, H2A, H2B, H3 and H4) [37,38]. In contrast, the GC protein Nucleostemin (GNL3), does not affect nucleolar localization of H2B-ECFP (Figure 1(e)). Of note, the localization of NLS-CFP in the nucleolus was unaltered upon Nucleolin knockdown (~95%) as compared to control cells (~96%) (Figure 4(c), Fig. S2A). In summary, Nucleolin is a key factor which modulates the localization of H2B-ECFP in the nucleolus.

Since Nucleolin knockdown reduced H2B-ECFP compartmentalization in the nucleolus, we performed the converse experiment of overexpressing Nucleolin. Interestingly, Nucleolin co-expression showed a consistent and enhanced retention of nucleolar H2B-ECFP in ~67% cells (24 h), which declined to ~42% (48 h), and ~24% (72 h) post transfection (Figure 4(d), black bars), while nucleolar H2B-ECFP declined rapidly over time from ~35% (24 h), ~10% (48 h) and ~5% (72 h) in control cells (Figure 4(d), white bars). Essentially, H2B-ECFP retention was significantly higher upon Nucleolin co-expression at each time point. Independently, NPM1 co-expression showed a moderate increase in nucleolar retention of H2B-ECFP (~57%, after 24 h), which was lower than upon Nucleolin co-expression (~67%) (Figure 4(d)). Taken together, Nucleolin regulates H2B-ECFP retention in the nucleolus.

We asked if the compartmentalization of H2B-ECFP in the nucleolus, correlates with endogenous levels of Nucleolin across cell lines (Figure 4(e–g)). Immunoblotting of whole cell extracts across cell lines showed an increase in Nucleolin levels as follows: CRL1790 < DLD1 < HCT116 < MCF7 (Figure 4(f)). Furthermore, increased nucleolar sequestration of H2B-ECFP positively correlates with an increase in the endogenous levels of Nucleolin in these cell lines (Figure 4(e,g)). We further corroborated this by overexpressing Nucleolin in DLD1 cells, which dramatically increased nucleolar compartmentalization of H2B-ECFP in ~67% cells, as compared to control cells (~40%) (Figure 4(g)). In summary, an increase in the endogenous or overexpressed levels of Nucleolin, positively correlates with the extent of H2B-ECFP in the nucleolus and Nucleolin therefore functions as a positive regulator of H2B-ECFP sequestration into the nucleolus.

### Nucleolin modulates H2B-ECFP dynamics in the nucleolus

**Figure 5.**
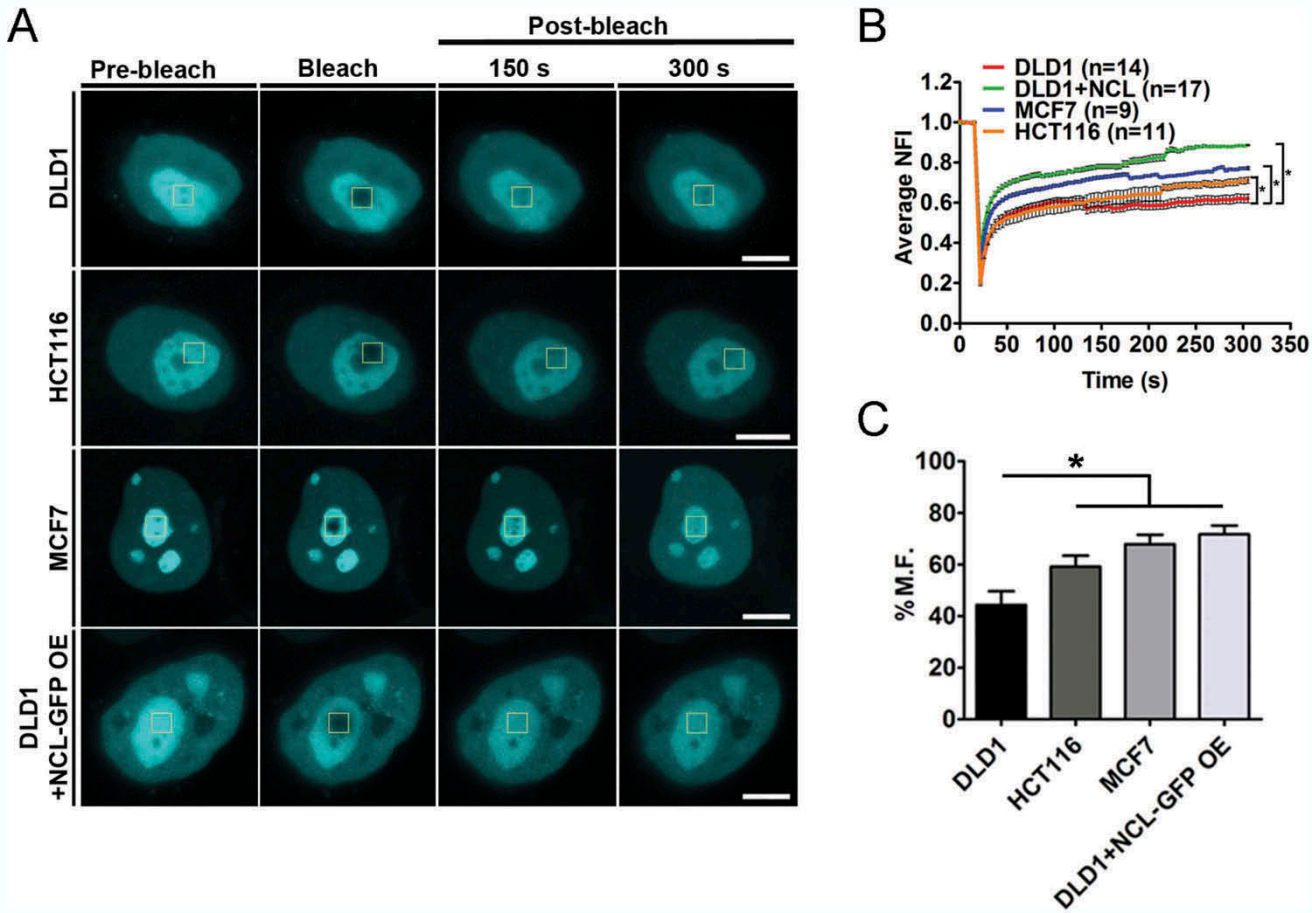
Nucleolin levels positively correlate with H2B-ECFP mobility. (a) FRAP of H2B-ECFP in the nucleolus of DLD1, HCT116, MCF7 and DLD1 cells co-transfected with NCL-GFP (DLD1+ NCL-GFP OE), Scale bar ~5 µm. (b) Normalized fluorescence recovery curves of H2B-ECFP in the nucleolus. (c) Relative mobile fractions of H2B-ECFP as calculated from (b), n = number of nuclei, data from N = 3 independent biological replicates, error bars: SEM in recovery curves and bar graph. Student’s t-test, *p < 0.05.

Nucleolin is a histone chaperone and evicts histones from DNA [19,39]. We monitored fluorescence recovery of labelled H2B in order to address the impact of Nucleolin on the mobility of H2B-ECFP (Figure 5(a,b)). Interestingly, H2B-ECFP showed a higher mobile fraction in HCT116 (M.F. ~59%) and MCF7 cells (M.F. ~68%) respectively, as compared to DLD1 cells (M.F. ~40%) (Figure 5(c)). Furthermore, DLD1 cells overexpressing Nucleolin showed a significantly higher mobility of H2B-ECFP in the nucleolus (DLD1 + NCL OE: M.F. ~72%) as compared to control cells (M.F. ~40%) (Figure 5(c)). Taken together, the mobility of H2B-ECFP in the nucleolus positively correlates with an increase in the levels of Nucleolin.

### Nucleolin interacts with H2B-ECFP

**Figure 6.**
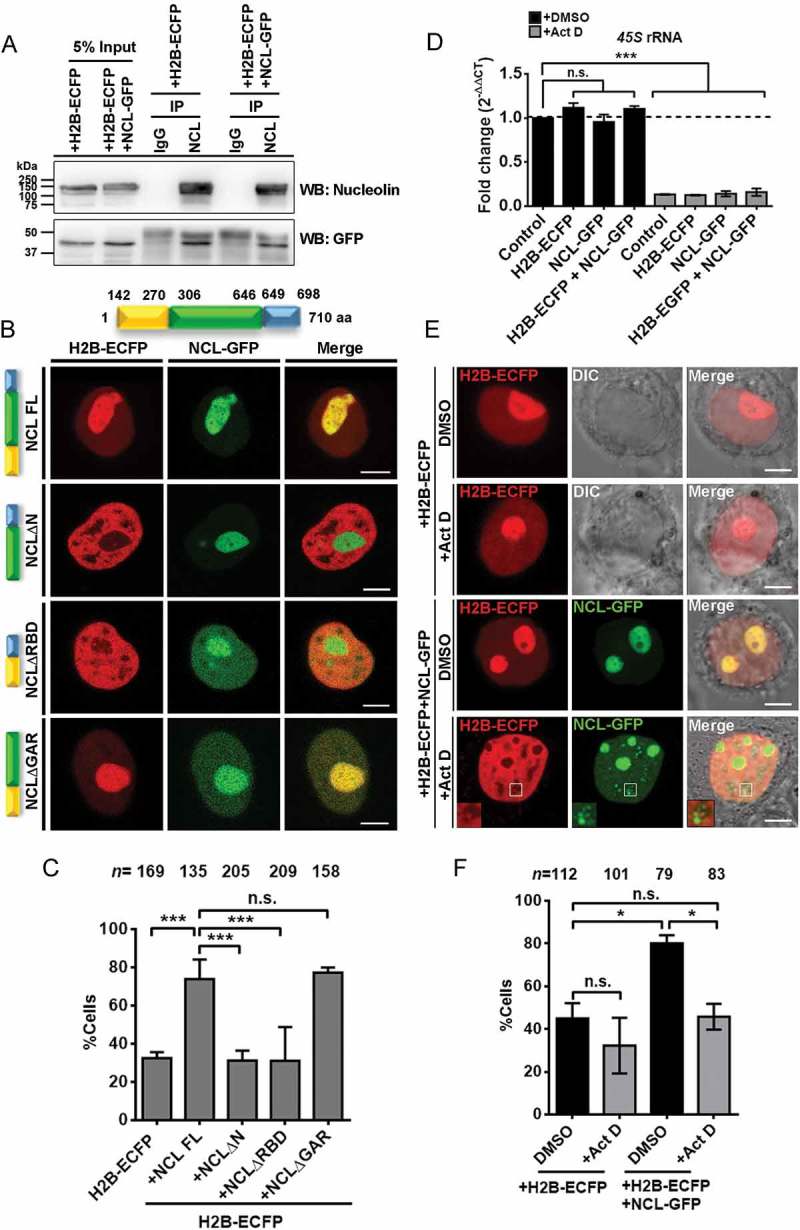
Nucleolin associates with H2B-ECFP in the nucleolus. (a) Co-immunoprecipitation (Co-IP) of endogenous Nucleolin and NCL-GFP with anti-Nucleolin antibody reveals its interaction with H2B-ECFP. Normal IgG used as a control for Co-IP. Anti-GFP antibody was used in western blot to detect H2B-ECFP. Data from N = 2 independent biological replicates. (b) Schematic showing three major domains of Nucleolin. The numbers denote the amino acid positions of the respective domains of Nucleolin. Representative images of DLD1 cells co-transfected with H2B-ECFP and Full length NCL (NCL FL), N-terminal deletion (NCLΔN), RNA binding domain deletion (NCLΔRBD) or GAR domain deletion (NCLΔGAR). H2B-ECFP and FL NCL show co-localization in the nucleolus. NCLΔRBD and NCLΔGAR show nucleoplasmic localization in addition to nucleolar localization. Scale bar, 5 µm. (c) Percent cells showing nucleolar H2B-ECP upon co-expression of NCL FL, NCLΔN, NCLΔRBD and NCLΔGAR, n = number of nuclei, data from N = 3 independent biological replicates, error bars: SEM. ANOVA, ***p < 0.001. (d) qRT-PCR for 45S pre-rRNA in vehicle (DMSO) and Actinomycin D (Act D, 0.05 μg/ml) treated cells, N = 2. 45S pre-rRNA level is downregulated in all cells upon Act D treatment. All statistics performed with respect to Control (+DMSO) cells, ANOVA, ***p < 0.001. (e) Representative images of cells expressing H2B-ECFP alone, or co-expressing NCL-GFP, upon DMSO or Act D treatment. Inset – Nucleolin speckles upon Act D treatment, do not show H2B-ECFP, Scale bar ~5 μm. (f) Quantification of percent cells showing nucleolar H2B-ECFP upon Act D treatment (from e), n = number of nuclei, N = 2 independent biological replicates. ANOVA, *p < 0.05.

Since Nucleolin modulates the retention and dynamics of H2B-ECFP in the nucleolus (Figures 4 and 5), we sought to examine if Nucleolin associates with H2B-ECFP. Remarkably, we found that H2B-ECFP co-immunoprecipitates with endogenous Nucleolin and independently with co-expressed NCL-GFP (Figure 6(a)). Furthermore, H2B-ECFP and NCL-GFP, co-localize in the nucleolus reiterating the association between Nucleolin and H2B-ECFP (Figure 6(b)).

We sought to investigate into the mechanisms of Nucleolin mediated sequestration of H2B-ECFP in the nucleolus. Towards this end, we examined the effect of co-expressing deletion mutants of Nucleolin into DLD1 cells and scored for H2B-ECFP compartments in the nucleolus. We co-expressed H2B-ECFP with (i) full length NCL FL (ii) NCLΔN (N-terminal deleted) (iii) NCLΔRBD (RBD1-4 deleted) and (iv) NCLΔGAR (GAR domain deleted). We observed a comparable localization of NCLΔN in the nucleolus as that of full length NCL, while NCLΔRBD and NCLΔGAR partially mislocalized in the nucleoplasm, consistent with previous studies (Figure 6(b)) [40–42]. Co-expression of full length NCL showed a significant increase in nucleolar H2B-ECFP (~74%), as compared to cells transfected with H2B-ECFP alone (~32%) (Figure 6(c)). Interestingly, co-expression of NCLΔN and NCLΔRBD did not enhance H2B-ECFP localization in the nucleolus, since both conditions showed ~31% cells with nucleolar H2B-ECFP (Figure 6(c)). In contrast, co-expression of NCLΔGAR showed a comparable extent of nucleolar H2B-ECFP as that of full length NCL (~77%) (Figure 6(c)). Taken together, the N-terminal and RNA binding domains of Nucleolin are essential for the enhanced localization of H2B-ECFP in the nucleolus.

### Nucleolin mediated nucleolar localization of H2B-ECFP is pre-rRNA dependent

Since NCLΔRBD did not enhance nucleolar H2B-ECFP localization, we determined if rRNA was necessary for NCL mediated localization of H2B-ECFP in the nucleolus. We treated DLD1 cells with Actinomycin D (0.05 µg/ml) for 4 hours, which showed a significant decrease in 45S rRNA levels (Figure 6(d)).

We asked if nucleolar localization of H2B-ECFP was affected upon inhibition of rDNA transcription by Act D treatment (Figure 6(e)). H2B-ECFP localized in the nucleolus in ~45% control cells (Figure 6(e,f)). This sub-population of cells marginally reduced upon Act D treatment (~32%) (Figure 6(e,f)). Co-expressing NCL-GFP enhanced nucleolar localization of H2B-ECFP in ~80% control cells (Figure 6(e,f)). However, upon Act D treatment, the NCL-GFP mediated increase in nucleolar H2B-ECFP (~77%) showed a significant reduction to ~46% (Figure 6(e,f)). This suggests a requirement of 45S rRNA in the sequestration of H2B-ECFP in the nucleolus.

It is noteworthy that upon Act D treatment, Nucleolin speckles in the nucleoplasm do not colocalize with H2B-ECFP (Figure 6(e), inset). However, Nucleolin shows a distinctive co-localization with H2B-ECFP in the nucleolus, in the presence of 45S pre-rRNA in the nucleolus. Taken together, Nucleolin and 45S rRNA are required for the compartmentalization of H2B-ECFP in the nucleolus.

## Discussion

### Overexpressed H2B localizes in the nucleolus

The nucleolus is a complex milieu of ribosomal DNA, RNA, proteins and non-ribosomal proteins [5]. Sequestration into the nucleolus is an important mode of post translational regulation of proteins such as ARF and Cdc14 that control cell cycle and apoptosis [43]. Histones and histone variants are commonly enriched in the nucleolus. The histone H1 variant H1.0, localizes in the nucleolus and is strongly associated with non-transcribed regions of ribosomal DNA and interacts with nucleolar proteins involved in rRNA processing [44,45]. Another histone variant – macroH2A also localizes at the nucleolus and is directly involved in rDNA repression [46]. Histone 2A methylated by Fibrillarin at Q104 in humans and Q105 in yeast, is exclusively localized in the nucleolus [47]. However, H2B transiently localizes in the nucleolus upon transfection and disperses into the nucleoplasm over time, either integrating or exchanging with nuclear chromatin [27]. In vitro, a higher concentration of histone octamers to DNA (>0.76 mass ratio), aggregates chromatin and inhibits transcription [48]. Furthermore, excess histone expression in budding yeast shows cytotoxicity and is deleterious to these cells [49,50]. We surmise, that the nucleolar sequestration of excess H2B, is a preferred paradigm for preventing the potentially deleterious effects of histone overexpression in the nucleus and toxicity across most cell types.

### Lamins as modulators of nuclear histone dynamics

Histones are hyperdynamic in ES cells which have a relatively open chromatin conformation [51]. Histone dynamics is dampened during differentiation and lineage commitment, as chromatin undergoes compaction. Lamin A/C levels are relatively lower in ES cells but increase during differentiation [52]. Consequently, Lamin A overexpression in ES cells, restricts histone H1 mobility [29]. Furthermore, Lamin B1 expression is lower in senescent cells with compact chromatin and Senescence Associated Heterochromatic Foci (SAHF) [53]. Lamin depletion in differentiated DLD1 cells, did not show an appreciable effect on H2B-ECFP dynamics in the nucleoplasm (Figure 2). This is consistent with relatively unaltered chromatin dynamics in differentiated cells upon masking of the histone binding domain of Lamin A/C [54]. We envisage the following scenarios of the role of Lamins in the modulation of histone dynamics – (1) Consistent with previous data, reduced expression levels of Lamin A/C or B-type lamins do not appreciably affect histone mobility in differentiated cells (Figure 2) [29,52] (2) It is likely that the combined depletion of Lamin A/C and B-type Lamins, alter histone mobility, in differentiated cell types (3) Lamin interactors such as Emerin, Lamin B receptor (LBR) and barrier to autointegration factor (BAF) with histone binding domains, maintain histone dynamics in the absence of Lamins [55,56].

On the other hand, Nucleostemin is highly expressed and is a marker of cancer stem cells [57]. Furthermore cancer stem cells show increased DNA accessibility as assessed by formaldehyde-assisted isolation of regulatory elements-sequencing (FAIRE-seq), suggesting open chromatin conformation [58,59]. We surmise that the decrease in H2B-ECFP mobility in the nucleoplasm upon Nucleostemin loss suggests reduced accessibility to chromatin in cancer stem cells. Interestingly, independent knockdowns of Lamin B1, Fibrillarin and Nucleostemin enhance H2B-ECFP mobility in the nucleolus (Figure 3). We surmise that Fibrillarin and Nucleostemin are bonafide nucleolar factors, that control the nucleolar microenvironment, as their depletion enhances H2B-ECFP dynamics to a significantly greater extent than nuclear lamin B1 (Figures 2 and 3). Furthermore, the loss of Fibrillarin, Nucleostemin or Lamin B1, potentially alter the relative stoichiometries of bound and unbound sub-fractions of H2B-ECFP with nucleolar chromatin and consequently enhance histone dynamics in the nucleolus [30,35,60].

### Nucleolin modulates H2B-ECFP localization into the nucleolus

Nucleolin exhibits a dominant role in sequestering H2B-ECFP into the nucleolus (Figure 4). Nucleolin is a high mobility group protein and is a major constituent of the granular component of the nucleolus [61]. Nucleolin is involved in rRNA transcription and processing [18,62]. Nucleolin is closely related to another nucleolar phosphoprotein – Nucleophosmin. Phase separation of Nucleophosmin and Fibrillarin to a relatively more viscous nucleolar phase is critical to the maintenance of nucleolar integrity [15,63]. In addition, ribosomal proteins – L3 and S3A and non-ribosomal proteins – Lamin B2 and HIV-rev, localize into the nucleolus by virtue of their interaction with Nucleolin and Nucleophosmin [9,31,64]. H2B is localized into the nucleolus through its nucleolar localization signal (NoLS) and electrostatic interaction with nucleolar components [27]. Here, we discovered the requirement of Nucleolin for the sequestration and retention of H2B-ECFP in the nucleolus. Nucleolin plays a more dominant role in the localization of H2B-ECFP in the nucleolus, since the loss of Nucleolin strikingly decreases nucleolar H2B-ECFP, while the co-expression of Nucleolin, retains H2B-ECFP in the nucleolus over a considerably longer duration (Figure 4). More importantly, the N-terminal domain, previously shown to interact with histones H1 and H2A-H2B dimers and the RNA binding domain of Nucleolin, are indispensable for the nucleolar retention of H2B-ECFP [21,22] (Figure 6). Taken together, the interaction between H2B-ECFP and Nucleolin in the nucleolus serves as a mechanism for the nucleolar localization and retention of overexpressed H2B.

### Nucleolin modulates nucleolar H2B-ECFP dynamics

Nucleolin levels modulate H2B-ECFP retention and dynamics in the nucleolus across cell types (Figures 4 and 5). Furthermore, the N-terminal domain and RBD of Nucleolin regulate H2B-ECFP compartmentation in the nucleolus (Figure 6). Nucleolin functions as a histone chaperone facilitating exchange of H2A-H2B dimers from chromatin [21,39]. However, nucleoplasmic and nucleolar H2B exist in distinct microenvironments. The nucleoplasmic pool of H2B largely associates with DNA, whereas nucleolar sub-pools of H2B reside in the microenvironment of nucleolar DNA, ribosomal RNA, non-coding RNAs such as snoRNAs, ribosomal and non-ribosomal proteins, which may collectively impinge on H2B dynamics in the nucleolus.

**Figure 7.**
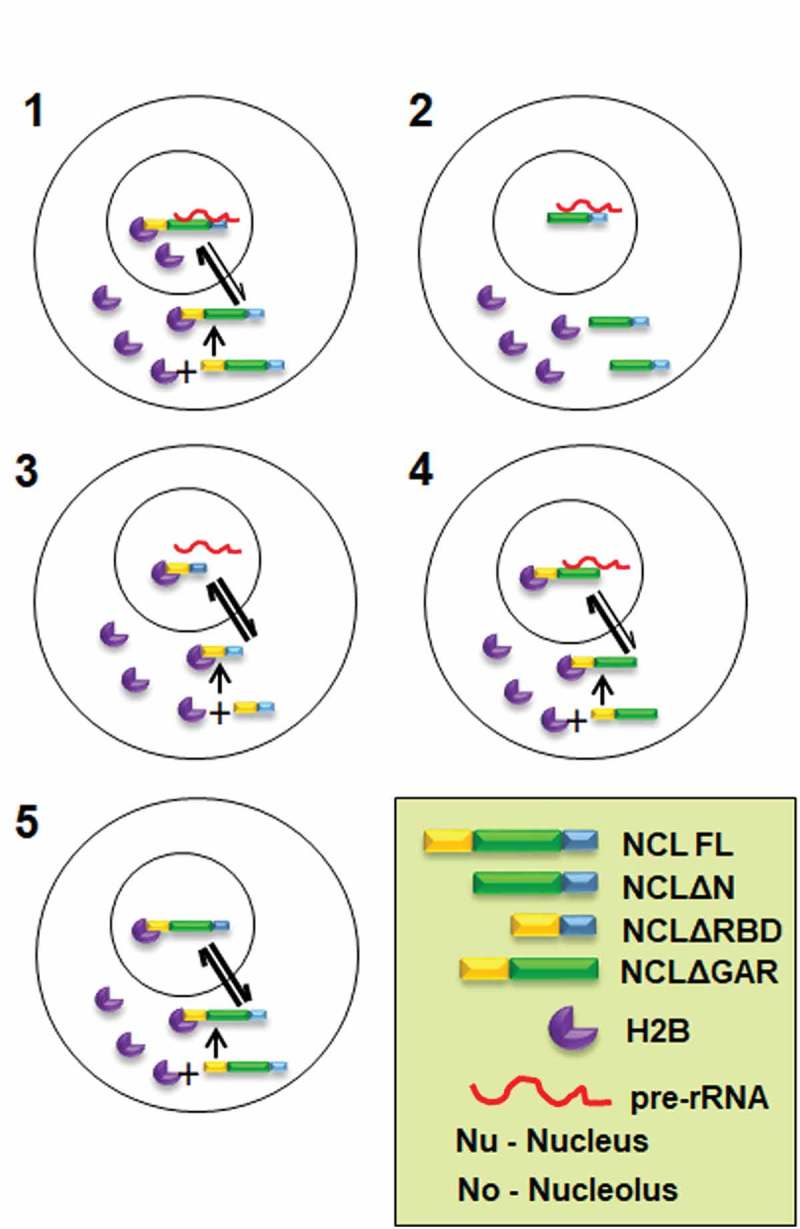
Speculative model of Nucleolin regulating nucleolar compartmentation and dynamics of H2B-ECFP. 1. Nucleolin interacts with H2B-ECFP via its N-terminal domain and shuttles it into the nucleolus. In the nucleolus, Nucleolin binds to pre-rRNA via its RNA binding domain and H2B-ECFP via its N-terminal domain, thus retaining H2B-ECFP in the nucleolus. It is likely that the relative rate of import of H2B-ECFP into the nucleolus is greater in the presence of Nucleolin. 2. In absence of the N-terminal domain, Nucleolin does not bind to H2B-ECFP, thereby reducing nucleolar pools of H2B-ECFP. 3. Nucleolin RBD deletion mutant binds to H2B-ECFP through its N-terminal domain and sequesters H2B-ECFP into the nucleolus. However, in the absence of RBD, H2B-ECFP is not retained in the nucleolus, as the RBD is required for binding to pre-rRNA. 4. GAR domain deletion mutant binds to H2B-ECFP and pre-rRNA and shows enhanced recruitment of H2B-ECFP into the nucleolus, similar to full length Nucleolin. 5. Nucleolin imports H2B-ECFP in the nucleolus but is unable to retain it in the nucleolus in the absence of pre-rRNA transcription inhibited by Act D.

We surmise that the N-terminal domain of Nucleolin rich in acidic amino acid stretches binds to nucleoplasmic H2B-ECFP and transports it to the nucleolus (Figure 7) [21,22]. Thus, with increased Nucleolin expression, there is enhanced H2B-ECFP import into the nucleolus, which correlates with an increase in the recovery of H2B-ECFP (Figure 5). We surmise that the enhanced retention of H2B-ECFP in the nucleolus upon NCL overexpression is also rRNA dependent. However, Act D treatment redistributes a sub-population of Nucleolin to the nucleoplasm, potentially resulting in the lowered retention of H2B in the nucleolus. The RNA binding domains of Nucleolin specifically interacts with the 5ʹ-ETS of pre-rRNA while GAR domain of Nucleolin non-specifically binds to any RNA [25,65]. In summary, the nucleolar retention of H2B-ECFP is dependent upon Nucleolin-45S rRNA complex. Therefore, the sub-domains of Nucleolin differentially affect nucleolar H2B-ECFP compartmentation. While the N-terminal domain is potentially required for translocating H2B-ECFP to the nucleolus, the RNA binding domain is also necessary for the retention of H2B-ECFP in the nucleolus.

### Implications

While it was previously proposed that overexpressed H2B localizes in the nucleolus, via charge based interactions between the positively charged H2B and the negatively charged nucleic acids within the nucleolar milieu, our studies for the first time unravel a novel Nucleolin guided mechanism that modulates the sequestration, retention and dynamics of H2B in the nucleolus [27]. This implicates Nucleolin, 45S rRNA and potentially other bonafide nucleolar factors namely Nucleophosmin in directing the fate of overexpressed and therefore excess nuclear proteins such as histones, into the nucleolus. Considering that the nucleolus is maintained as discrete phase separated entities in the nucleus, the mechanisms involved in targeting nuclear factors into or out of the nucleolus are largely unclear [12]. Histone gene expression is tightly regulated and coupled to DNA replication during S-phase [50,66]. Imbalances in histone expression and its accumulation can induce G1 cell cycle arrest, genomic instability and affect transcription [67–69]. It is therefore conceivable that Nucleolin/Nucleophosmin are specifically involved in dual roles of chaperoning out excess nuclear proteins such as histones into the nucleolus. Furthermore, this study also unravels the key involvement of ribosomal RNA as an essential mediator that facilitates the retention of nucleolar H2B. A combination of nucleolar factors and their interaction with rRNA is potentially involved in the generation of the phase separated nucleolus – a unique non-membranous milieu within the nucleoplasm, for the rapid but regulated entry and exit of factors that potentially facilitate rRNA biogenesis. In summary, this study unravels a unique and novel mechanism whereby proteins are guided and retained into phase separated systems such as the nucleolus. This suggests potential implications towards the targeted therapeutic intervention of dysregulated ‘cancer nucleoli’.

## Materials and methods

### Plasmids

H2B-ECFP [70], GFP-nucleolin [71], GFP-NPM1 and NLS-CFP plasmids were kind gifts from Jennifer Lippincott-Schwartz, Sui Huang and Tom Misteli, respectively. NCLΔN, NCLΔRBD and NCLΔGAR plasmids were generated from GFP-nucleolin by restriction free (RF) cloning. Primers used for RF cloning are as follows: NCLΔN: Sense, 5ʹ-GCAAGAATGCCAAGAAGCCTGTCAAAGAAGCACC-3ʹ, Antisense, 5ʹ-GGTGCTTCTTTGACAGGCTTCTTGGCATTCTTGC-3ʹ; NCLΔRBD: Sense, 5ʹ-GAACCGACTACGGCTAAGGGTGAAGGTGGC-3ʹ, Antisense, 5ʹ-GCCACCTTCACCCTTAGCCGTAGTCGGTTC-3ʹ; NCLΔGAR: Sense, 5ʹ-CTGGGCCAAACCTAAGGACCACAAGCCACAAG-3ʹ, Antisense, 5ʹ-CTTGTGGCTTGTGGTCCTTAGGTTTGGCCCAG-3ʹ.

### Cell lines, cell culture and transfections

DLD1 colorectal adenocarcinoma cells (Gift from Thomas Ried, NCI/NIH) were grown in RPMI medium (Gibco, 11875), HCT116 colorectal carcinoma and MCF7 breast adenocarcinoma (ATCC) cells were grown in DMEM (Gibco, 11995), CRL1790 normal colon cells were grown in MEM (Gibco, 11095). All media were supplemented with Penicillin (100 units/ml)/Streptomycin (100 μg/ml) (Gibco, 15070-063) and 10% heat inactivated FBS (Gibco, 6140). Cells were cultured at 37°C in the presence of 5% CO_2_. We authenticated cell lines by karyotyping metaphases derived from each of these cell lines. We routinely tested cells in culture and found them free of mycoplasma contamination. Transient transfections were performed using Lipofectamine RNAimax reagent and siRNA (100 nM) (Invitrogen, 13778) in reduced serum medium OptiMEM (Gibco, 31985) for 6 h, after which cells were transferred to complete medium and incubated for a total duration of 48 h at 37°C. The siRNA oligonucleotide sequences were – FBL: 5ʹ-AGGAGAACAUGAAGCCGCA-3ʹ, FBL Scramble: 5ʹ-GAAGAACGAUCAGGACAAU-3ʹ; GNL3: 5ʹ-GCUUAAAACAAGAACAGAU-3ʹ, 5ʹ-AUGUGGAACCUAUGGAAAA-3ʹ; GNL3 Scramble: 5ʹ-AUAAUCGAACGAUAAGAAC-3ʹ; LMNA/C: 5ʹ-CAGUCUGCUGAGAGGAACA-3ʹ;LMNA/C Scramble: 5ʹ-GGAGGUCGAGCCAAUAUCA-3ʹ; LMNB1: 5ʹ-AGACAAAGAGAGAGAGAUG-3ʹ; LMNB1 Scramble: 5ʹ-GAGGGAAACGUAAAGAAGA-3ʹ; LMNB2: 5ʹ-GAGCAGGAGAUGACGGAGA-3ʹ; LMNB2 Scramble: 5ʹ-GGAAGCGUAGACGGAAGAG-3ʹ. NCL: 5ʹ-UCCAAGGUAACUUUAUUUCUU-3ʹ; NCL Scramble: 5ʹ-GCUAGCUUUAUUCGUAUAUUA-3ʹ, NPM1: 5ʹ-AGATGATGATGATGATGAT-3ʹ. Transient plasmid transfections were performed using Lipofectamine LTX with Plus reagent (Invitrogen, 15338-100) and cells were imaged after 24 h of transfection at 37°C. For FRAP analyses upon knockdowns, siRNA and DNA transfections were performed sequentially – siRNA transfection was performed as mentioned previously, while DNA transfections were performed after 24 h and cells were imaged by fluorescence microscopy, after 48h of siRNA transfection at 37°C.

### Western blot, antibodies and co-immunoprecipitation

SDS–PAGE and immunoblotting were performed according to standard protocols. Lysates were prepared in RIPA buffer, protein concentration was estimated using bicinchoninic acid (BCA) kit (Pierce, 23225), resolved on SDS-PAGE and transferred to Immobilon-P polyvinylidene difluoride (PVDF) membranes (Millipore, IPVH00010) for 90 min at 90 volts. Membranes were blocked with 5% non-fat dried milk in Tris-buffered saline, 0.1% Tween-20 (TBST) for 1h at Room Temperature (RT). Primary and secondary antibodies were diluted in 0.5% non-fat dried milk in 1X TBST. Primary antibodies: anti-Nucleolin (Abcam, ab22758), 1:1000; anti-Actin (Abcam, ab3280), 1:400; anti-histone 2B (Millipore, 07-371, gifted by Sanjeev Galande), 1:1000; anti-Fibrillarin (Abcam, ab4566), 1:1000; anti-Nucleostemin (Abcam, ab70346), 1:2000; anti-Lamin A/C (Epitomics, S2526), 1:5000; anti-Lamin B1 (Abcam, ab16048), 1:1000; anti-Lamin B2 (Abcam, ab8983), 1:400; anti-GFP (Abcam, ab290), 1:1000 for 3h at RT or overnight at 4°C. Secondary antibodies: Sheep anti-Mouse-HRP (Amersham, NA9310V), 1:10,000; Donkey anti-Rabbit-HRP (Amersham, NA9340V), 1:10,000, for 1h at RT. Between incubations, membranes were washed three times in 1X TBST for 10 minutes each at RT. Immunoblots were developed using chemiluminescent substrate ECL Prime (Amersham, 89168-782) and imaged with ImageQuant LAS4000. Relative levels of H2B and Nucleolin were quantified from western blots using Image J (http://imagej.nih.gov/ij/). The intensity of the band was normalized to the respective loading controls.

For co-immunoprecipitation (Co-IP) assays, ~10^7^ cells (DLD1) were lysed in co-IP lysis buffer (50 mM Tris [pH 7.4], 150 mM NaCl, 0.5% NP-40, 1× PIC) vortexed and incubated on ice for 15 min, and centrifuged at 12,000 rpm and 4°C for 10 min. The lysate was precleared by incubating with Dynabeads protein A (Invitrogen, 10002D) for 1 h. Anti-Nucleolin antibody (ab22758, 3 µg) or normal rabbit IgG was incubated with lysates overnight at 4°C. Protein A beads, pre-blocked with 0.5% BSA, were incubated with the immunocomplex for ~3 h. Beads were washed 6 times with co-IP lysis buffer to minimize non-specific binding. Bound protein was eluted from the beads by boiling in 2× Laemmli buffer for 15 min at 95°C. Samples were resolved on a 15% SDS-PAGE, followed by western blotting.

### qRT-PCR

RNA was prepared by lysing cells in TRIzol (Invitrogen) followed by phenol-chloroform extraction, cDNA was synthesized using the Verso cDNA synthesis kit (Thermo Fisher Scientific). Semi-quantitative real-time PCR (qRT-PCR) was performed using SYBR green (SAF labs). *ACTIN* served as internal control. The primers used for qRT-PCR were:

*45S*: Forward, 5ʹ-GAACGGTGGTGTGTCGTT-3ʹ; Reverse, 5ʹ-GCGTCTCGTCTCGTCTCACT-3ʹ and *ACTIN*: Forward, 5ʹ- GATTCCTATGTGGGCGAC-3ʹ, Reverse: 5ʹ-GGTAGTCAGTCAGGTCCCG-3ʹ.

### Immunofluorescence assay

Cells grown on coverslips (18 × 18 or 22 × 22 mm^2^) were briefly washed twice using 1X Phosphate Buffered Saline (PBS) and fixed for 10 min in 4% Paraformaldehyde (Sigma, P6148) in PBS, pH 7.4 at RT, washed thrice in 1X PBS (5 min each), followed by permeabilization in 0.5% Triton-X-100 (in PBS) for 10 min at RT. Cells were blocked in 1% Bovine serum albumin (BSA) (Sigma, A2153) in 1X PBS, for 30 min, washed thrice with 1X PBS and incubated in primary antibodies for 90 min at RT and secondary antibodies for 60 min at RT, with washes in between using 1X PBS. Primary antibody was diluted in 0.5% BSA (prepared in 1X PBS): anti-Nucleolin (ab13541), 1:300, anti-H2B (Millipore, 07-371), 1:100. Following secondary antibodies were used after diluting in 1X PBS +0.1%Triton X-100 (PBST): anti-mouse IgG-Alexa 568 (Molecular Probes), 1:1000; anti-rabbit IgG Alexa 488 (Molecular Probes) after which cells were washed thrice in 1X PBST. Cells were mounted in Slowfade Gold Antifade (Invitrogen, S36937). Cells were imaged on a Zeiss LSM710 confocal microscope with 405 nm, 458 nm and 561 nm laser lines at 2% power using a 63X oil immersion objective, NA 1.4 at 2.5X digital zoom. X-Y resolution: 512 pixels X 512 pixels (1 pixel = 0.11 µm). Confocal z-stacks were collected at an interval of 0.32 µm.

### Fluorescence Recovery after Photobleaching (FRAP) experiments and analysis

A Zeiss LSM710 confocal microscope equipped with a heated stage at 37°C, was used for all photobleaching experiments and fluorescence image acquisitions. For live imaging, cells were grown on a 22 × 22 mm^2^ coverslip glued onto a 35 mm petridish coated with 100 μg/ml Collagen (BD Biosciences, 354236), CO_2_ independent Leibovitz L-15 medium (Gibco, 21083-027) supplemented with 10% FBS (complete L15), was used during microscopy. Images were acquired using a 63X oil immersion objective, NA 1.4 at 2.5X digital zoom, at 2% laser power to avoid photobleaching. The acquisition parameters were adjusted to avoid bleed-through of ECFP and GFP fluorescence. A 10 pixel X 10 pixel square (1 pixel = 0.11 µm) Region of Interest (ROI) was bleached in both nucleoplasmic and nucleolar H2B-ECFP. Photobleaching was performed using the 405 nm laser line at 100% power. Laser iterations of 120 and 150 were used to photobleach labelled H2B in the nucleus and nucleolus respectively. Images were collected every 3.87 s for a total duration of 5 min. Images were analyzed using Zen 2011 FRAP Analysis module and normalized fluorescence intensity (NFI) was calculated as follows:

(1) $$\begin{array}{rl} & NFI=\left[ROI1\left(t\right)-ROI3\left(t\right)\right]/\left[ROI2\left(t\right)-ROI3\left(t\right)\right]\\ & X\left[ROI2\left(t=0\right)-ROI3\left(t=0\right)\right]\\ & \;\;\;\;\;\;\;\;\;\;\;\;\;\;\;\;\;\;\;\;\;\;\;\;\;\;\;\;\;\;\;\;\;\;\;\;\;\;\;\;\;\;\;\;/\;\;\left[ROI1\left(t=0\right)-ROI3\left(t=0\;\right)\right] \end{array}$$

where, *ROI1* is the fluorescence intensity of the 10 px X 10 px ROI that is bleached, *ROI2* is the whole nucleus fluorescence intensity and *ROI3* is the fluorescence intensity of a 10 px X 10 px background region selected outside the nucleus. *ROI1(t)*: post-bleach fluorescence intensity at time t. *ROI2(t)* and *ROI3(t)*: whole nucleus and background, respectively. *ROI1(t = 0)*: average pre-bleach fluorescence intensity. *ROI2(t = 0)* and *ROI3(t = 0)*: whole nucleus and background, respectively. The NFI was plotted as a function of time to generate double normalized FRAP curves.

Mobile fractions of H2B-ECFP were calculated as follows:

(2) $$\%Mobile\;fraction=\left(Ffinal-Fbleach)/(Fprebleach\;\;\;\;\;\;\;\;\;\;\;\;\;\;\;\;\;\;\;\;\;\;\;\;-Fbleach\right)\;\times \;100$$

Where, *Ffinal* is the NFI at maximum recovery, *Fbleach* is the NFI at the instant of bleaching and *Fpre-bleach* is the NFI before bleaching.

### Actinomycin D treatment

Cells transfected for 24 h were treated with 0.05 μg/ml actinomycin D (Act D) in complete medium for 4 h at 37°C with 5% CO_2_ after which they were transferred to complete L-15 medium and imaged live. Equivalent volumes of dimethyl sulfoxide (DMSO) were used as vehicle controls.

### Cell cycle analyses

Cells were fixed in 70% ethanol (in 1X PBS) and subjected to RNase treatment and propidium iodide staining for 1 hour at 37°C. Cells were scanned on FACS Calibur (BD Biosciences). Cell cycle analyses was performed by ModFit software.

### Statistical analysis and graphs

Two-tailed student’s t-test was used to compare the number of cells showing nucleolar H2B-ECFP compartments and mobile fractions of H2B-ECFP, p-value <0.05 was considered significant. Graphs were plotted using GraphPad Prism software.
