# Supplementary figures and images for: Nucleolin modulates compartmentalization and dynamics of histone 2B-ECFP in the nucleolus
Source: Nucleus. 2018 Jun 26;9(1):350–67. doi: 10.1080/19491034.2018.1471936 (PMC6165600; doi:10.1080/19491034.2018.1471936)

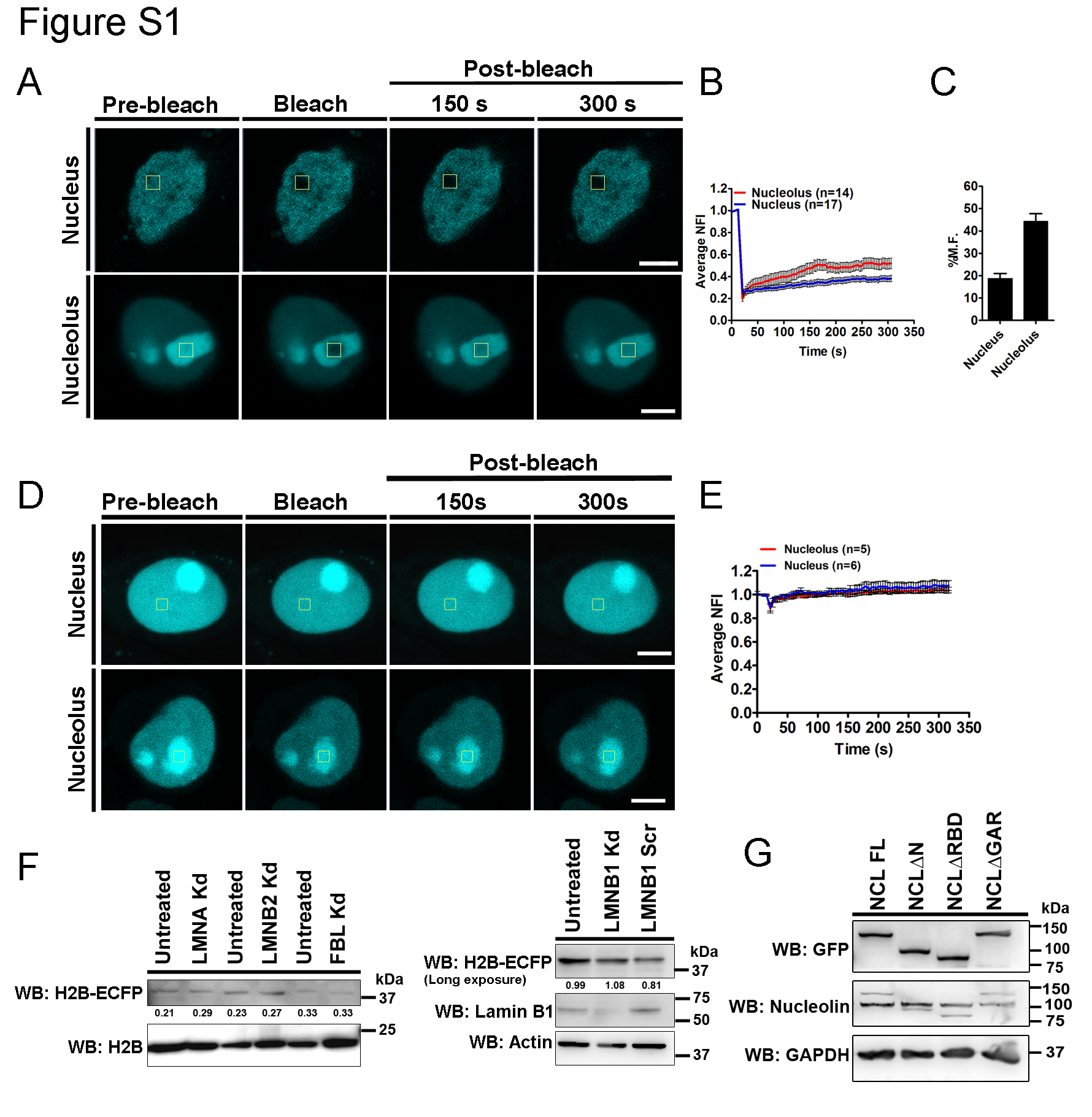

Supplement: Supplemental Material [file kncl-09-01-1471936-s001.zip › Supplementary information/Figure S1.tiff]

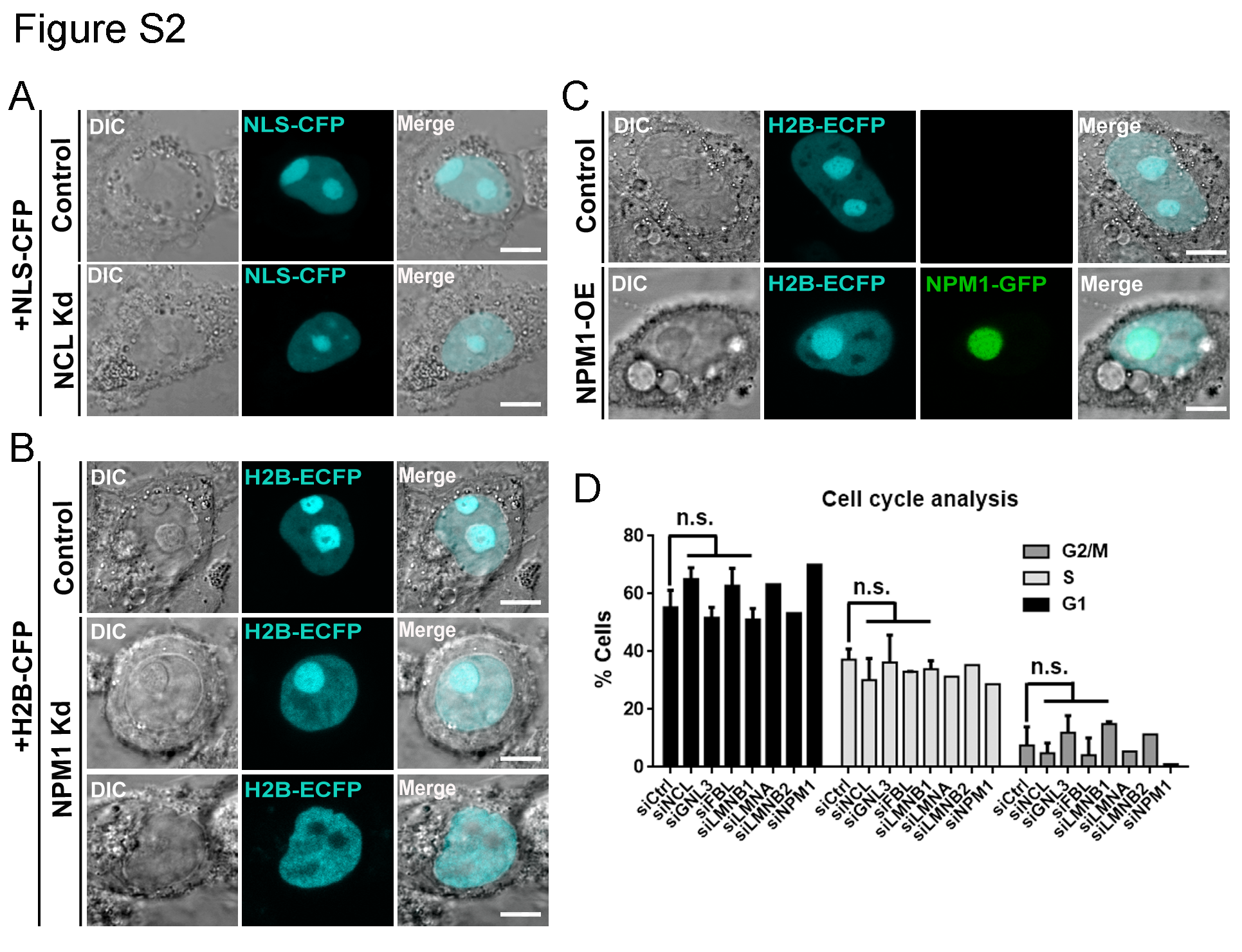

Supplement: Supplemental Material [file kncl-09-01-1471936-s001.zip › Supplementary information/Figure S2.tif]
